# PD-L1 testing, fit for routine evaluation? From a patholo﻿gist’s point of view

**DOI:** 10.1007/s12254-016-0292-2

**Published:** 2016-10-28

**Authors:** Georg Hutarew

**Affiliations:** Department of Pathology, University Hospital and Paracelsus Medical University Salzburg, Müllner Hauptstraße 48, Salzburg, 5020 Austria

**Keywords:** Immune checkpoint inhibitors, PD-1, PD-L1, Immunohistochemistry, Biomarker assay

## Abstract

Tumours with high somatic mutation rates escape immune surveillance by upregulating receptors and ligands such as programmed death receptor-1 and its ligand (PD-1/PD-L1). Checkpoint inhibitors (ICI) provide encouraging therapeutic results in non-small cell lung cancers (NSCLC) and may soon be used in 2nd or 1st line therapy. Currently PD-L1 immunohistochemistry (IHC) expression assessed on tumour cells is used as a predictive biomarker, since better patient outcomes are often, but not always associated with increased tumour cell PD-L1 IHC expression. However pre-analytical variables, different anti-PD-L1 clones used on different staining platforms, different specimens types, as well as intra- and interobserver variability influence the results. We will only understand PD-L1 expression on tumour cells if we accept that PD-L1 is an inducible pathophysiological factor with variable levels of PD-L1 expression depending on the immunological status. Should we test PD-L1 during initial diagnostic work up before, or at the point when immune checkpoint therapy is considered? Taking all arguments into account the value of PD-L1 as a predictive biomarker is questionable. Other predictive biomarkers such as high mutation burden, mRNA expression, neo-antigens and the diversity of tumour antigen-specific T cells should be evaluated in the future. Here we review results presented in 30 journal articles and three reviews covering this topic in the last 3 years.

## Immune receptors and ligands

Within their microenvironment tumour cells can modify the immune response, which comprises a dynamic balance of multifactorial interactions with stimulating and inhibitory receptors and ligands of immune cells. The same mechanisms help create immune tolerance and prevent autoimmune diseases [1]. Particularly tumours with a high rate of somatic mutations such as lung cancers or melanomas are immunogenic [2–5]. They induce upregulation of receptors and ligands such as the programmed death receptor-1 and its ligand (PD-1/PD-L1) and B7/CTLA-4, and consequently they escape immune surveillance. Checkpoint inhibitors, especially monoclonal antibodies against PD-1 and its ligand PD-L1 have proven to provide a promising therapeutic approach in non-small cell lung cancer (NSCLC) [1, 6–10].

## PD-L1 IHC expression as a predictive biomarker

**Fig. 1:**
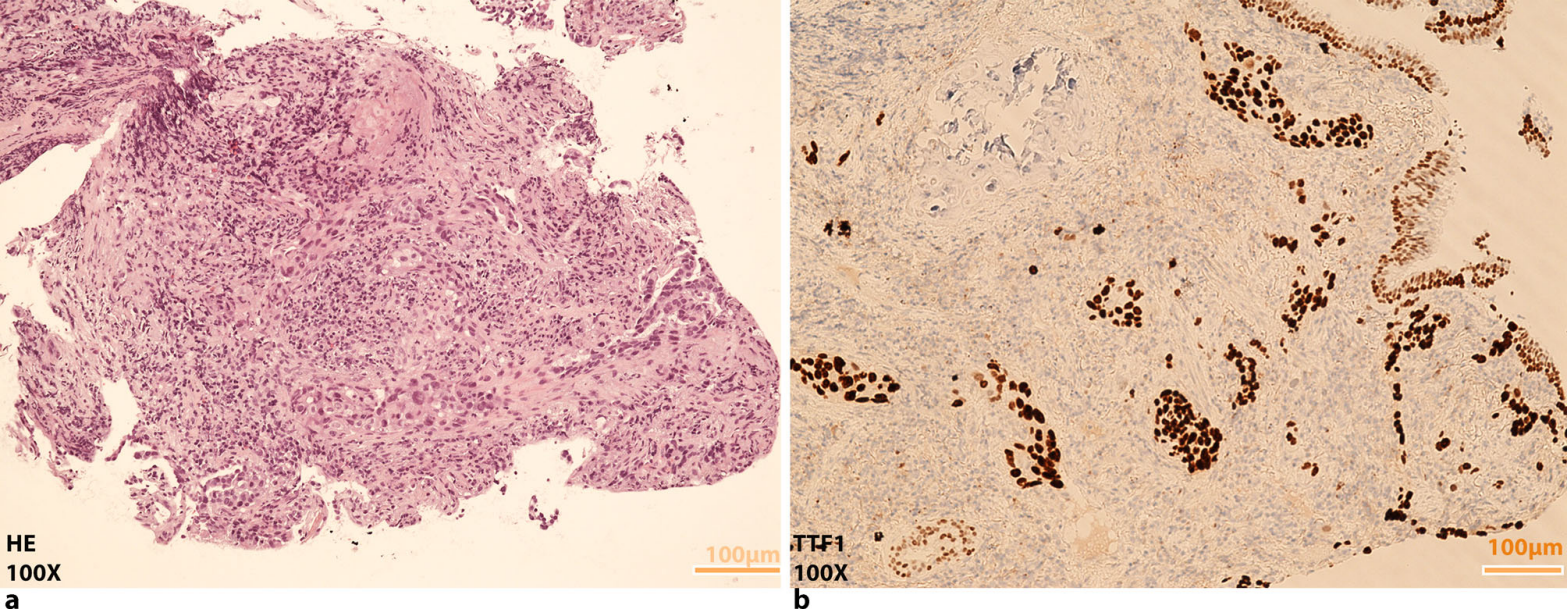
Pulmonary adenocarcinoma. **a** HE and **b** positive TTF1 staining, both images ×100 magnification

**Fig. 2:**
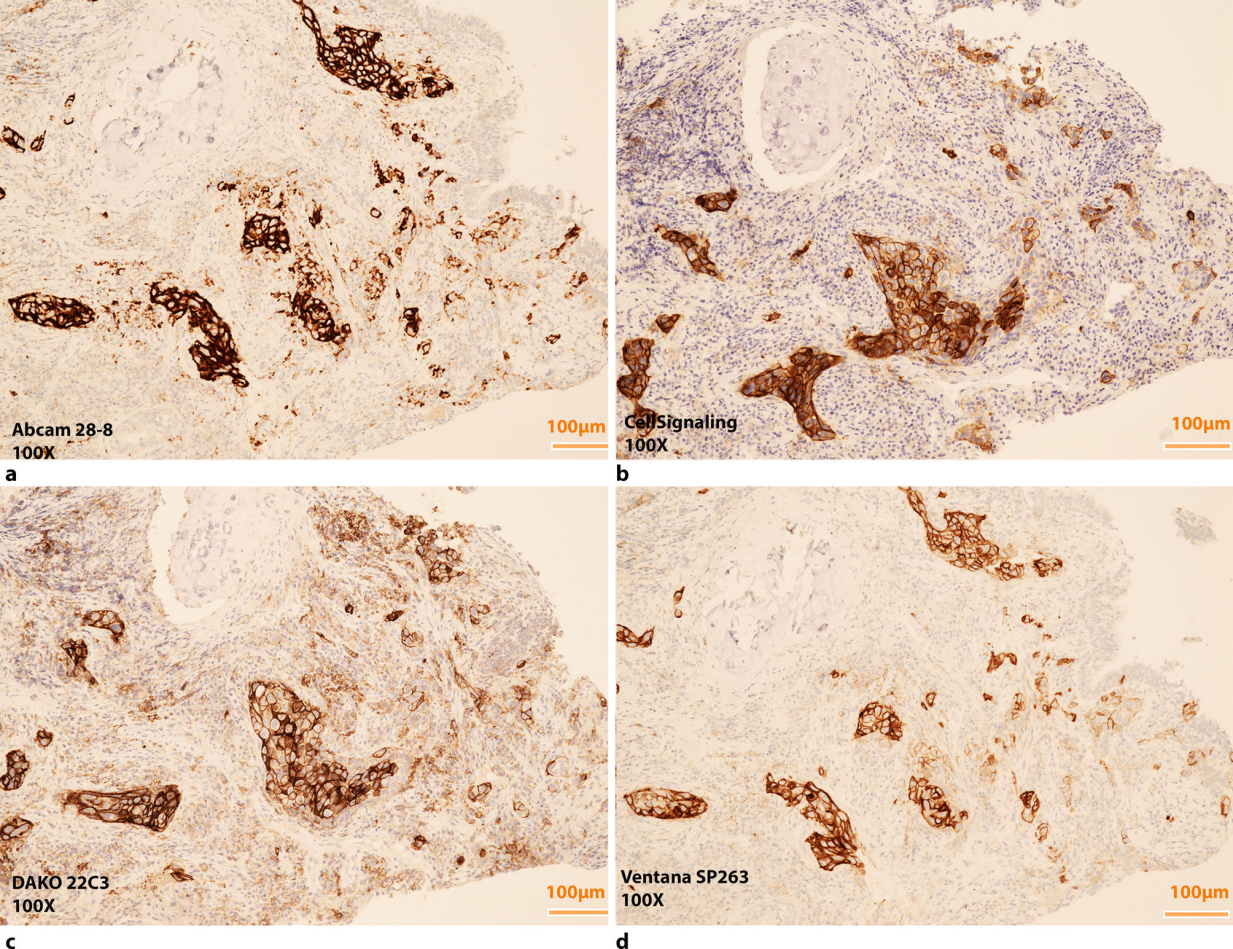
Images of the TTF1 + pulmonary adenocarcinoma seen in Fig. 1 stained positive with different PD-L1 antibody clones, Cross-testing, (**a,b**) Abcam 28-8 and Cell Signaling E1L3N and, both stained on Ventana Ultra with OptiView, (**c,d**) DAKO Pharm DX 22C3 and Ventana SP263, both prepackaged kits. Scoring does not evaluate intensity therefore enhancement systems such as OptiView can be used without altering the results. All images ×100 magnification

Today numerous PD-1 inhibitors are available such as nivolumab (Opdivo, Bristol Myers Squibb, NY, USA) and pembrolizumab (Keytruda, MSD, Kenilworth, NJ, USA) or PD-L1 inhibitors such as durvalumab (AstaZeneca, London, UK), atezolizumab (Roche, Basel, Switzerland) and avelumab (Pfizer/Merck Serono, Berlin/Darmstadt, Germany). Since checkpoint inhibitors may be used in 2nd or 1st line therapy in the near future, it is important to define a reliable predictive biomarker. One candidate is the expression of PD-L1 assessed by immunohistochemistry (IHC) especially on tumour cells [5, 7]. A number of PD-L1 antibody clones are available as prepackaged kits or as free antibodies, using different staining platforms and different staining protocols as well as different scoring systems and different cut offs for predictive evaluation (Table 1; [7]). They can be used on Ventana Ultra Systems, DAKO Autolink 48 stainers or others with or without enhancement systems (Figs. 1 and 2).

**Table 1:** Specifications of PD‑L1 Antibody Clones

Antibody clone/test	Source and clonality	Cdx for drug	Mechanism	Cutpoint	Staining system	Compartment
VENTANA PD-L1 (SP263)^b^	Rabbit monoclonal	Durvalumab (AstraZeneca)	Anti PD-L1	TC ≥ 25 %	Ventana Ultra	Tumour cell membrane
PD-L1 IHC 28-8 pharmDx Dako^b^	Rabbit monoclonal	Nivolumab (BMS)	Anti PD-1	TC ≥ 1 %	Dako Autolink 48	Tumour cell membrane
28-8 Abcam^b^	Rabbit monoclonal	–	–	TC^c^	Multiple platforms	Tumour cell membrane
PD-L1 IHC 22C3 pharmDx Dako^b^	Mouse monoclonal	Pembrolizumab (Merck USA)	Anti PD-1	TC ≥ 1 %	Dako Autolink 48	Tumour cell membrane
22C3 DAKO^b^	Mouse monoclonal	–	–	TC^c^	Multiple platforms	Tumour cell membrane
PD-L1/CD274 (SP142) Spring^b^	Rabbit monoclonal	Atezolizumab (Roche)	Anti PD-L1	TC and/or IC see Table 2	Ventana Ultra	Tumour cell membrane and Immune cells
E1L3N Cell signaling^a^	Rabbit monoclonal	–	–	TC^c^	Multiple platforms	Tumour cell membrane
CAL10 Biocare Medical^a^	Rabbit monoclonal	–	–	TC^c^	Multiple platforms	Tumour cell membrane

^a^Some clones are only available as free antibodies and ^b^others as free antibodies and as kits

^c^depending on the ICI used

*PD‑L1* programmed death ligand, *ICI* immune checkpoint inhibitor, *TC* tumour cells, *IC* immune cells

## Is there an optimal threshold?

The results of “The Blueprint PD-L1 IHC Assay Comparison Project”, a phase I study, were presented by Dr. Hirsch et al. at the recent 2016 Annual Meeting of the AACR. In this study three of four assays were analytically similar for tumour cell staining, i. e. SP263, 28-8 and 22C3, but no clinical diagnostic cut-off was applied in the project. At the same AACR meeting, M. Ratcliffe presented “A Comparative Study of PD-L1 Diagnostic Assays”. Evaluating 500 biopsy samples including both squamous and non-squamous histology and showed that a 25 % cut-off point using Ventana SP263 was similar to the results obtained from a DAKO 28-8 test at 10 % cut-off mark. The results from the SP263 and the Dako 22C3 tests were similar at a cut-off of 50 %. All three tests agreed overall in more than 90 % of cases.

In a review by Kerr et al. [6], the calculated rate of positivity in 10 analysed studies for PD-L1 was between 13 and 70 %[11–14] and the correlation between treatment and biomarker response rate was given as 13–83 % depending upon the cut-offs, the specific antibody clones as well as the therapeutic agent used [7, 15]. Tumour proportion scores (TPS) were defined as the percentage of tumour cells with complete or partial membranous staining at any intensity. A wide range of cut-off points determined IHC positivity with values of 1, 5, 10, 25 and 50 % [11, 16–18]. Commonly high expression of PD-L1 indicates a better therapy response [5, 7, 13, 16, 19–23] and showed improving hazard ratios for overall survival (OS) and progression free survival (PFS) with increasing levels of PD-L1 staining [24]. Nevertheless many studies also report significant response rates (3–20 %) in PD-L1 IHC-negative cases [6, 12, 15, 25].

Khunger et al. presented “Meta-analysis of tumour PD-L1 expression as a predictive biomarker of benefit from PD-1/PD-L1 axis inhibitors in solid tumours” at ASCO 2016. The analysis evaluated 18 studies with 2731 patients. Inclusion criteria were different tumours with high mutational burden, with 9 studies of NSCLC with known IHC PD-L1 status and PD1/PDL1 inhibitor treatment. A threshold of 5 % PD-L1 IHC expression was highly predictive for different drugs as nivolumab, pembrolizumab, atezolimumab, durvalumab and avelumab. The largest therapeutic effect was seen in NSCLC (OR = 3.33; 95 % CI 2.52–4.40, *p* < 0.001). The authors concluded that 5 % tumour PD-L1 expression as a threshold of PD-L1 expression may be optimal.

Results of the phase III CheckMate057 study [12] showed that PD-L1 IHC with a cut-off point of 1 % correlated with ORR and PFS in pretreated NSCLC. Likewise Passiglia et al. [15] calculated a threshold of 1 % for PD-L1 expression based on evaluating 7 studies with 914 patients with PD-L1 positive tumours. These patients had a significantly higher ORR, than patients with PD-L1 negative tumours (OR: 2.44; 95 % CI 1.61–3.68) [15].

Kerr et al. reported the use of different thresholds in different biomarker studies using the example of nivolumab [1, 5]. Trials of this agent used anti–PD-L1 IHC antibody clone 28-8 (Dako, Glostrup, Denmark) with cut-off points of ≥1, ≥5 and ≥10 % to define positive staining [13]. Eventually, nivolumab was approved without the need for complementary diagnostic. Biomarker tests for pembrolizumab used anti–PD-L1 Dako clone 22C3 with two different cut-offs of ≥1 % and ≥50 % in the conducted study to define positive staining and for clinical use, ≥50 % TPS was considered positive [20, 26, 27]. Meanwhile pembrolizumab has been approved by the EMA with a cut-off point of 1 % with any approved IHC test similar to 22C3. Durvalumab uses the anti–PD-L1 Ventana SP263 antibody clone with a cut off of ≥25 % [25].

## PD-L1 testing on immune cells and/or tumour cells

Azetolizumab with SP142 Ventana as a companion diagnostic requires assessment of TC and/or tumour-associated immune cells (ICs) (Table 2; [23]).

**Table 2:** PD-L1 scoring convention for TC and IC expression detected by Ventana SP 142

Description PD-L1 staining (%)	IC score	Description PD-L1 TC staining (%)	TC score
IC ≥ 10	IC3	TC ≥ 50	TC3
IC ≥ 5 and <10	IC2	TC ≥ 5 and <50	TC2
IC ≥ 1 and <5	IC1	TC ≥ 1 and < 5	TC1
IC < 1	IC0	TC < 1	TC0

*IC* immune cells, *TC* tumour cells

Herbst et al. [4] showed that PD-L1 expression on tumour infiltrating immune cells predicts responses to atezolizumab better than PD-L1 expression on tumour cells. Teng et al. reported that combining IHC PD-L1 expression status of tumour-infiltrating lymphocytes and tumour cells might help to select patients for combination therapies [5, 28].

Biomarker expression in lymphoid or other immune effector cells is a special challenge for pathologists. Inter- and intra-observer bias for TILs is lower than for tumour cells [28], the pathologist cannot always recognize whether the existing lymphocyte population is oncogene or inflammation driven [23, 28]. Scheel et al. showed that reproducible PD-L1 IHC scoring of tumour cells seems feasible whereas scoring of immune cells did not yield reproducible results [29].

The threshold that discriminates between therapy responders and nonresponders should be calculated from collected response data; however it is not clear whether benefit from immunotherapy might be better described by progression-free or overall survival data than by overall response rate [3, 10, 16, 20]. Studies suggest that traditional response criteria may not be able to fully capture the immune-therapy activity [15].

## Does heterogeneity of lung cancer influence PD-L1 results?

PD-L1 expression results might not represent the true PD-L1 status of a tumour due to the heterogeneity of PD-L1 expression in lung cancer, this is especially challenging if only biopsies are evaluated [1]. Kerr and other authors [1, 5, 16] stated that in a multifactorial dynamic system reacting sensitive to changes, any earlier form of chemotherapy or targeted therapy may induce PD-L1 expression. The opposite was reported by Herbst et al. in abstract 3030 ASCO 2016 “Archival vs new tumour samples for assessing PD-L1 expression in the KEYNOTE-010 study”. The distribution of PD-L1 (TPS 1–49 % and >50 %) was similar in archival and newly collected tumour samples of patients with previously treated NSCLC. These data suggest that a new tumour biopsy sample may not be required at the time when ICI therapy is considered, questioning the value of rebiopsy.

## Does histology affect PD-L1 testing?

Patients with squamous cell carcinomas treated with nivolumab did not show improvements in PFS and OS dependent on the level of PD-L1 expression [3]. In patients with non-squamous cell carcinoma treated with nivolumab or docetaxel, the nivolumab treated group with positive PD-L1 IHC expression showed better PFS and OFS [12]. Hence mainly tumours with non-squamous histology should be tested for PD-L1 expression in lung cancer.

## Standardizing PD-L1 testing

PD-L1 tests must be reproducible, both the technical procedure of staining and the interpretation of the test by pathologists. Pre-analytical issues such as tissue fixation and processing have a major impact on the outcomes of immunohistochemical reactions [14, 21] and might affect the results of different PD-L1 IHC tests.

Standardization of these biomarker tests can be reached by using exclusively prepackaged test kits of reagents running on company-specific staining platforms with an industry standard [24]. However free antibody clones such as Abcam 28-8, Cell signalling E1L3N and others are less expensive than their prepackaged counterparts and can be established on different staining platforms without quality impairment. The results obtained from cross testing PD-L1 IHC 28-8 pharmDx on Autostainer Link 48 versus Anti-PD-L1 antibody 28-8 (Abcam) on Ventana Ultra with OptiView in our department showed no significant differences in staining results (unpublished data). Worldwide numerous cross-assay validations and interlaboratory tests (round robin tests) are performed to determine the reproducibility of PDL1 immunohistochemistry yet without having found a gold standard.

## Other predictive biomarkers

Upcoming biomarkers may include the high mutation burden within the PD-L1 positive tumour cell group, expression of neo antigens, the diversity of the T cell repertoire and PD-L1 mRNA expression [30].

## Conclusion

PD1/PD-L1 biology is complex with conflicting results from different studies. Moreover PD-L1 IHC does not fulfil the strict criteria of a biomarker as anaplastic lymphoma kinase (ALK) translocation or epidermal growth factor receptor (EGFR) mutation does. Nevertheless PD-L1 IHC expression seems to be the best currently available biomarker and may be indicative of a dose–response relationship between PD-L1 expression and drug efficacy. A low threshold such as a PD-L1 TPS of 1 % allows us to include nearly all patients who may really benefit from these therapies. However, since it may be inappropriate to select patients for ICI therapy solely on the basis of PD-L1 expression other predictive biomarkers should be established. In advanced lung cancer plasma PD-L1 protein could provide a promising alternative for monitoring PD-L1 levels. PDL1-enzyme linked immunosorbent assay (PDL1-ELISA) can analyse PDL1 quantitatively or qualitatively in plasma and PDL1 western blot might help to detect specific proteins in tissue homogenate. Mutational findings from targeted NGS panels can be correlated with response, but until today targeted NGS panels were not able to predict response to checkpoint inhibitors. Looking at DNA only provides limited information therefore, if we understand mRNA as a molecule reflecting the dynamic nature of a cancer cell, we should focus on investigating the cancer transcriptome in future.
